# Effectiveness of rib fixation compared to pain medication alone on pain control in patients with uncomplicated rib fractures: study protocol of a pragmatic multicenter randomized controlled trial—the PAROS study (Pain After Rib OSteosynthesis)

**DOI:** 10.1186/s13063-022-06509-0

**Published:** 2022-09-02

**Authors:** Jean Yaniss Perentes, Michel Christodoulou, Etienne Abdelnour-Berchtold, Wolfram Karenovics, Angèle Gayet-Ageron, Michel Gonzalez, Thorsten Krueger, Frédéric Triponez, Philippe Terrier, Benoît Bédat

**Affiliations:** 1 Unit of Thoracic and Endocrine Surgery, Centre Hospitalier Universitaire Vaudois, Lausanne, Switzerland; 2 Unit of Thoracic and Endocrine Surgery, Hospital of Valais, Sion, Switzerland; 3 Unit of Thoracic and Endocrine Surgery, University Hospitals of Geneva, Rue Gabrielle-Perret-Gentil 4, 1205 Geneva, Switzerland; 4 CRC & Division of Clinical-Epidemiology, Department of Health and Community Medicine, University of Geneva & University Hospitals of Geneva, Geneva, Switzerland; 5 Haute-Ecole Arc Santé, HES-SO University of Applied Sciences and Arts Western Switzerland, Neuchâtel, Switzerland

**Keywords:** Rib fractures, Rib osteosynthesis, Pain, Chronic pain

## Abstract

**Background:**

Persistent pain and disability following rib fractures result in a large psycho-socio-economic impact for health-care system. Benefits of rib osteosynthesis are well documented in patients with flail chest that necessitates invasive ventilation. In patients with uncomplicated and simple rib fractures, indication for rib osteosynthesis is not clear. The aim of this trial is to compare pain at 2 months after rib osteosynthesis versus medical therapy.

**Methods:**

This trial is a pragmatic multicenter, randomized, superiority, controlled, two-arm, not-blinded, trial that compares pain evolution between rib fixation and standard pain medication versus standard pain medication alone in patients with uncomplicated rib fractures. The study takes place in three hospitals of Thoracic Surgery of Western Switzerland. Primary outcome is pain measured by the brief pain inventory (BPI) questionnaire at 2 months post-surgery. The study includes follow-up assessments at 1, 2, 3, 6, and 12 months after discharge. To be able to detect at least 2 point-difference on the BPI between both groups (standard deviation 2) with 90% power and two-sided 5% type I error, 46 patients per group are required. Adjusting for 10% drop-outs leads to 51 patients per group.

**Discussion:**

Uncomplicated rib fractures have a significant medico-economic impact. Surgical treatment with rib fixation could result in better clinical recovery of patients with uncomplicated rib fractures. These improved outcomes could include less acute and chronic pain, improved pulmonary function and quality of life, and shorter return to work. Finally, surgical treatment could then result in less financial costs.

**Trial registration:**

ClinicalTrials.govNCT04745520. Registered on 8 February 2021.

## Introduction

### Background

Until recently, persistent pain and disability after uncomplicated rib fractures have been poorly documented [1]. A recent cohort study described persistent pain and disability in, respectively, 59% and 76% of patients at 2 months [2], and in 22% and 53% of patients at 6 months [3]. Shelat et al. reported that 23% of patients had chronic pain one year after simple rib fractures. In a retrospective study including 216 patients with an isolated thoracic injury, only 34.2% of patients had a good recovery at 1 year and the 6-month return to work rate is of 63% [4]. A recent analysis of 1074 trauma patients admitted to an emergency department [5] highlighted a linear relationship between the number of fractured ribs and length of stay in the hospital and opioid use. Persistent pain and disability following rib fractures therefore could result in a large psycho-socio-economic impact for health-care system.

The only predictive factor for persistent pain and disability is the pain intensity within the first few days after injury [2, 3]. Similarly, the intensity of pain within the first days after thoracotomy predicts long-term post-thoracotomy pain [6]. In a recent meta-analysis, epidural analgesia provided better acute pain relief than intravenous, paravertebral, and intercostal interventions [7].

While a recent meta-analysis concluded that operative fixation of complicated flail chest provided a better outcome [8], the impact of surgery on pain in case of uncomplicated rib fractures is seldom studied. Some retrospective studies showed promising results of rib fixation with surgery in patients with uncomplicated rib fractures: De Moya et al. showed that rib fixation reduced postoperative analgesic requirements [9]. Similarly, Wu et al. showed that pain was significantly reduced 1 month after surgery as compared to a non-surgical approach [10]. Fitzgerald et al. showed a decreased mortality and respiratory complications after surgery in patients over 65 years old as well as a better functional status at 2 weeks, 2 months, and 4 months [11]. More recently, a clinical trial with 23 patients randomized showed a benefit with lower pain in the rib fixation group as compared to the nonoperative group at 2 weeks [12].

### Trial objectives

We aim at bringing further knowledge to improve care of trauma patients with uncomplicated rib fractures. Indeed, no previous studies have provided definitive evidence for recommending rib fixation over simple pain medication to control pain in these patients. Given the high prevalence of chronic pain in patients with isolated rib fractures [3], there is an urgent need to clarify whether rib fixation could improve patients’ outcomes. Our hypothesis is that a surgical approach may have further benefits regarding pain control and consecutive complications and disabilities as compared to a conservative treatment. Primary objective is to compare pain 2 months after injury between group (1) patients receiving rib fixation and standard analgesic treatment and group (2) patients treated with standard analgesic treatment alone. Secondary objective is to assess the amount of pain medication, the quality of life, the occurrence of anxiety and depression symptoms, the pulmonary capacity, the return to work, and adverse events of treatments received within 1-year post-injury. Medical costs will also be investigated.

## Methods/design

### Trial design

The PAROS (Pain After Rib OSteosynthesis) trial is a pragmatic multicenter, randomized, superiority, controlled, two-arm, not-blinded trial that compares pain evolution between rib fixation and standard pain medication to standard pain medication alone in patients with uncomplicated rib fractures.

### Setting

The study will take place in three hospitals of Western Switzerland (French-speaking part). The *Hôpitaux Universitaires de Genève (HUG)* is the public hospital of the Canton of Geneva. The *Centre Hospitalier Universitaire Vaudois (CHUV)* is the main public hospital of the Canton de Vaud. The hospital of Sion, which is part of *the Centre Hospitalier du Valais Romand*, is the main public hospital of the Canton du Valais (Wallis). Tertiary care units specialized in thoracic surgery will host the study. Each center hospitalizes approximately 60–120 patients per year with uncomplicated rib fractures. These units are used to working together through an inter-hospital structure (Center for Thoracic Surgery of Western Switzerland).

### Participants

Study’s participants will be recruited among patients admitted at the local emergency room with uncomplicated rib fractures. To optimize enrolment, the staff of emergency departments of each study site will receive information and regular updates about the trial. Initial care management for rib fractures in the emergency department will not be modified to reflect the current situation. The screening process and the selection of participants will be performed by an attending surgeon from local surgical thoracic unit, under the responsibility of the local principal investigator. Reasons for exclusion, as well as the number of subjects who decline participation, will be documented.

Demographic data and comorbidities (Charlson Comorbidity Index [13]) will be recorded. Inclusion and exclusion criteria are detailed in Table 1. Main inclusion criteria are the presence of epidural analgesia and at least two uncomplicated rib fractures. According to the most recent local hospital guidelines, epidural analgesia is systematically proposed to reduce pain and is most often applied in the absence of contraindications. Therefore, only patients under epidural analgesia will be included, with the advantage to obtain a homogeneous sample of participants regarding acute analgesic treatment.

**Table 1:** Eligibility criteria

Inclusion criteria	Exclusion criteria
• At least 2 rib fractures • At least 1 dislocated rib fracture • Fractures accessible to surgery • Thoracic trauma no more than 2 days prior to screening for inclusion • Thoracic epidural analgesia • Written informed consent	• Any other concomitant fractures excepted clavicle fracture • Respiratory distress syndrome according to the Berlin definition [14] • Presence of >1.5 l of blood drained from the pleural space • Hemostasis disorder defined by any of the following criteria: - Platelet count < 70,000/mm^3^, - International normalized ratio (INR) > 1.2 (prothrombin < 70%) - activated partial thromboplastin time (aPTT) ≥ 60 s - drugs such as: P2Y12 antagonists (clopidogrel, prasugrel) and glycoprotein IIb/IIIa antagonists (abciximab, tirofiban) • Pathological rib fracture due to metastasis • Hemodynamic instability: systolic blood pressure < 100 mmHg and heart rate > 100 beats per minute • Neurologic disorder: Glasgow Coma Score < 13 in the initial 24 h, or intracerebral, epidural, subdural, or subarachnoid hemorrhages, or cerebral contusion • Titanium allergy • Known or suspected non-compliance to medical therapy due to drug or alcohol abuse • Age <18 years old • Women known to be pregnant or breast feeding thus contraindicating surgery • Inability to follow the procedures of the study, e.g., due to language problems, psychological disorders, dementia, etc.

### Assignment of interventions and blinding

The R package *blockrand* [15] will be used to generate the allocation sequences independently from study collaborators. Group allocation (1:1 ratio) will be performed using center-stratified block randomization procedure with random permuted block sizes (4, 6, 8). Then, participants’ allocation to groups will be performed using REDCap (Research Electronic Data Capture [16]) hosted at HUG. A comprehensive guide for performing randomization with REDCap is available online [17]. Practically, the procedure will go as follows: first, informed consent will be obtained by the site PI or his delegate from patients who meet eligibility criteria (Table 1). Inclusion will be confirmed by the local PI. Then, baseline observations will be carried out during the day following the inclusion and the group allocation will be obtained through REDCap. Given the nature of the intervention, the participants and the care management team will not be blinded to group membership (open trial).

### Surgery

Surgical procedures (rib fixation) are carried out during a maximum of five days after hospitalization. It is thought that surgery performed as soon as possible would have more impact on initial pain. To control the infectious risk, an antibiotic prophylaxis (Cefuroxime 1.5g IV) will be administered 30 min before surgery. Rib fixation will be performed by a senior surgeon under general anesthesia. A thoracotomy focused on the fracture will be performed to optimize the access to the broken rib. Video-assisted thoracic surgery (VATS) can be performed before the osteosynthesis to better localize rib fractures. During thoracotomy, significant muscle division is avoided. Removal of the periosteum is not required. The broken rib segments are approximated with forceps and the medical devices are used to fix the fracture. The medical devices are implemented according to the manufacturers’ recommendations. The goal is to stabilize the chest wall. It is not useful to fix all fractures to stabilize the wall. A chest tube can be placed at the end of the operation. The following medical devices can be used:

- MatrixRIB™, De Puy Synthes Companies, Zuchwill, Switzerland
- STRATOS™, MedXpert GmbH, Heitersheim, Germany
- NiTi Fixing Plates^TM^, IAWAI, Yandzhou, China

They are already used in the three study centers in patients with rib fractures. However, there is no evidence that the intervention being tested will be superior to medical therapy. The choice of the type of medical devices will be let to the appreciation of the surgeon. MatrixRIB is usually used. However, Stratos system and NiTi Fixing Plates appear to be easier to apply behind the scapula. The devices will be sterilized before use following standard surgical procedures and packaged by the sterilization unit at each hospital. The supply of the product is managed by the hospitals according to the standard procedures. The type, batch number, and length of the implanted materials will be documented.

### Standard pain control treatments

After inclusion and until discharge, the study’s participants will continue to be taken in charge in the surgical thoracic units at each center. Medical treatment and analgesia used after the randomization will be applied following the written procedure. Epidural analgesia will be continued for 1 to 7 days post-randomization to maximize outcome benefits. Afterwards, paracetamol, NSAID (non-steroidal anti-inflammatory drugs), and/or opioid treatment will be prescribed according to pain severity. In the case of opioid use, morphine treatment will be preferred. However, other opioid drugs or doses can be considered to better customize the treatment. Patients allocated to the control group will be monitored until discharge. Discharge criteria are no thoracic drain, acceptable pain level, and no intravenous medication.

### Outcomes

The primary outcome is pain level, assessed via the first part of the French version of the brief pain inventory (BPI) questionnaire measured at 2 months post-surgery. The BPI has become one of the most widely used measurement tools for assessing clinical pain [18, 19]. Pain severity score is obtained by averaging the individual scores (worst pain, least pain, average pain, current pain) coded on a 0–10 scale. Pain assessment will also be longitudinally performed at months #1, #2, #3, #6, and #12.

Secondary outcomes will be:

- *Amount and type of analgesic medication.* During hospitalization, each day since the enrolment, amount and type of analgesic medication will be recorded from the computerized patient record system. After discharge, analgesic medication will be tracked using a custom questionnaire. If any, the opioid amount will be recorded in equivalent dose of analgesics.
- *Anxiety and depression.* In order to highlight anxiety and depression symptoms, the Hospital Anxiety and Depression Scale (HADS) questionnaire will be applied at baseline and at 2 months [20]. HADS score lies between 0 and 21, with 21 indicating depression or anxiety.
- *Neuropathic pain.* The French questionnaire “*Douleur Neuropathique en 4 Questions* (DN4)” will be completed by the clinical research assistant or the surgeon during the follow-up visit at 2 months. The DN4 questionnaire was developed to diagnose polyneuropathy. Three items are linked with neuropathic pain examination, and seven items to pain symptoms, with a final score between 0 and 10. A score higher than four indicates neuropathic pain. The DN4 questionnaire has been broadly used and validated [21].
- *Pain interference and quality of life.* The last items of the BPI questionnaires measure how much pain has interfered with seven daily activities, including general activity, walking, work, mood, enjoyment of life, relations with others and sleep. BPI pain interference is scored as the mean of the seven items coded on a 0-10 scale. To assess quality of life, health, and wellbeing, the short-form health survey (SF-36 [22]) will be used, including 8 dimensions: physical functioning, bodily pain, limitations due to physical health problems, limitations due to personal or emotional problems, emotional well-being, social functioning, energy/fatigue, and general health perceptions All questions are scored on a scale from 0 to 100, with 100 representing the highest level of functioning possible. Items of a specific area are then averaged together, for a final score [0-100] within each of the eight dimensions [23].
- *Productivity impairment and return to work.* The work productivity and activity impairment (WPAI) questionnaire is an instrument to measure impairments in both paid work and unpaid work. It measures absenteeism, presenteeism as well as the impairments in unpaid activity because of health problems during the past 7 days [24]. The final score lies between 0 and 10 with higher values indicating higher impairment.
- *Pulmonary function and respiratory muscle function.* Forced vital capacity (FVC, % predicted), peak expiratory flow (PEF, L/min), and sniff nasal inspiratory pressure and maximum inspiratory pressure (in cmH2O) will be measured according to the recommendations of the European Respiratory Society [25]. The best value of at least three tests will be recorded.
- *Length of hospital stay.* Length of hospital stay will be reported in days from the day of hospitalization until hospital discharge.
- *Medical direct costs.* Costs are reported for each patient at the end of the follow-up period (12 months) in Swiss francs and will include costs for hospital care, surgery, and all related medications and drugs.
- *Adverse events.* We will collect safety outcomes in accordance with international standards (ISO 14155 [26] and ICH-GCP [27]).

### Measurement procedures

The schedule for recording the study’s outcomes is shown in Table 2. At hospital, sociodemographic information, as well as medication and daily pain, will be obtained from the patient’s medical record. Baseline measures will be collected at bedside by the local investigator or his/her delegate; questionnaires will then be filled in onto the web interface of the electronic case report form (eCRF). Special care will be taken to not influence participants when filling the questionnaires.

**Table 2:** Participant timeline

		Study period						
	Enrolment	Allocation	Post-allocation and discharge	Follow-up				Close-out
Timepoint	***−1D***	T0	4D	***1M***	***2M (visit)***	***3M***	***6M (visit)***	***12M***
**Enrolment:**								
**Eligibility screen**	X							
**Informed consent**	X							
**Allocation**		X						
**Interventions:**								
**Rib fixation**			X					
**Assessments:**								
**Pain and pain interference (BPI)**	X			X	X	X	X	X
**Anxiety and depression (HADS)**	X			X	X			X
**Neuropathic pain (DN4)**					X			
**Quality of life (SF-36)**	X				X		X	X
**Analgesic medication**	X			X	X	X	X	X
**Productivity and return to work (WPAI)**				X	X	X	X	X
**Pulmonary function**					X		X	
**Length of hospital stay**			X					
**Total costs**								X

Timepoints are given in days (D) and months (M) with T0 at allocation time

*BPI* brief pain inventory, *HADS* Hospital Anxiety and Depression Scale, *DN4* neuropathic pain diagnostic questionnaire, *SF-36* short form (36) health survey, *WPAI* work productivity and activity impairment questionnaire

After discharge, web surveys will be used to collect questionnaires. Phone calls can replace web surveys for participants without an internet connection. During visits (at 2 and 6 months after injury), pulmonary function and respiratory muscle function will be measured.

### Statistical methods

Baseline characteristics will be described by randomization group: continuous variables by their mean and standard deviation (SD), and median (interquartile range, IQR); categorical variables by their frequencies and relative percentages. Primary outcome will be compared between intervention and comparator groups using analysis of covariance ANCOVA [28] with BPI at 2 months as a dependent variable, randomization group as the main independent variable, baseline BPI, and sites as adjustment factors. Adjusted mean BPI-score difference between the intervention and controlled group will be reported with its 95% confidence interval (95% CI).

Primary analysis will be performed on the intention-to-treat population (as-randomized) and completed by per-protocol analysis after exclusion of major deviations from the protocol (patients hospitalized during more than 5 days and randomized initially in the intervention group but who will not be operated due to over-delay, or, on the contrary, patients in the control group who would be operated for medical reasons). Secondary analyses which consist in longitudinal modeling will be performed using mixed linear models to assess the evolution of pain (BPI) over time then anxiety and depression (HADS) score, quality of life, and finally productivity impairment (WPAI); an interaction between the time-points and the randomization group will be investigated. For secondary outcomes measured once (DN4, pain interference, length of stay, and total costs), we will perform non-parametric Mann-Whitney tests comparing both randomization groups, due to expected skewed distributions of those measurements. Finally, adverse event occurrence will be described according to the randomization groups. In addition, subgroup analyses will be performed to report the treatment effect by investigation sites and then by types of medical devices.

### Sample size

The sample size was calculated for an ANCOVA, according to results of previous studies [1, 29]. We aim to detect a difference of at least 2 on the BPI scale (μ2−μ1=2) between rib fixation and medical analgesia at 2 months. Considering a SD of 2 [1], a significance level of 0.05 (two-sided), and a power set at 90%, we calculated that 46 patients per group will be needed. Anticipating 10% dropouts, 51 patients per group will be required. The following conditions of participants will be considered as withdrawal from the study (loss to follow-up): voluntary withdraw or failure to stick to the protocol (moved away, became unable to communicate, missing or deceased).

### Confidentiality

Medical information obtained as a result of this study is considered confidential and disclosure to third parties is prohibited. All electronic files and databases are secured with password-protected access systems. Participant’s records in the eCRF are identified by code number. On each study site, written forms, or any other listings, are stored in a locked file with limited access. The project principal investigator (sponsor-investigator) will have direct access to his own site data sets and will have access to other site data only through the eCRF. Data communicated to project team members are blinded of identifying participant information.

For data verification purposes, authorized representatives of the sponsor-investigator, a competent authority, or the ethics committee may require direct access to parts of the medical records relevant to the study, including participants’ medical history.

### Monitoring and auditing

Independent monitoring is performed by the CRC (Clinical Research Center) of the University Hospitals of Geneva. Monitoring activities are performed according to ISO 14155 [26]. The monitors verify that regulatory documents are complete and up to date. Participating centers are visited on-site by a skilled monitor. Five visits are planned. The monitors review the source documents to determine whether the data reported in the web-based system are complete and accurate. Specific attention is paid to the primary outcome, informed consents, and serious adverse events. All findings and comments are documented in a monitoring report and communicated to sponsor-investigator, who initiates corrective action if necessary.

The ethics committee may wish to conduct an audit during the study or even after its completion. The sponsor-investigator and the investigators in the participating sites will support the inspectors in their activities.

### Protocol amendments and dissemination policy

Modifications to the protocol affecting important aspects of the study (objectives, design, target population, sample size, intervention procedure, and changes in local investigators), or affecting patient safety will require a formal amendment approved by the ethics committee. Minor changes, such as minor corrections and/or clarifications that have no effect on the way the study is conducted are documented and notified to the ethics committee.

The sponsor-investigator assures that all results of this study will be published in a peer-reviewed journal. All results relate to all outcomes as defined in this protocol regardless of statistical significance. The present article and other future articles follow the principles of the SPIRIT [30] and CONSORT [31] guidelines. Co-authorship on any of the publications will be based on contribution to the study and manuscript according to the criteria of the International Committee of Medical Journal Editors. No professional writer will be hired.

## Discussion

The PAROS trial compares pain control at 2 months after surgical versus non-surgical treatment among patients with uncomplicated rib fractures. Uncomplicated rib fractures have a significant medico-economic impact. Surgical treatment with rib fixation could result in better clinical recovery of patients with uncomplicated rib fractures. These improved outcomes could include less acute and chronic pain, improved pulmonary function and quality of life, and quicker return to work. Finally, surgical treatment could then result in less financial costs.

To the best of our knowledge, this is the first multicenter randomized controlled trial to evaluate pain at 2 months as a primary outcome in patients suffering from uncomplicated rib fractures. Three hospitals in Switzerland participate in this trial.

### Risk-benefit balance

Rib fixation is a low-risk procedure. A known adverse effect after rib fixation is hardware infection. A retrospective study among 122 patients operated for rib fractures with a MatrixRIB™ system and RibLoc® plating System showed 4.1% of hardware infection [32]. Outcomes were favorable after an antibiotic treatment and hardware retrieval. The other potential adverse effects that could require hardware retrieval are hardware intolerance or breaking of the plate or splint. However, the rate of complications is low, and the risks associated to rib fixation seem to be low as compared to the risk of persistent pain after rib fractures. Therefore, it is expected that the clinical benefits of rib fixation (a decreased pain) counterbalance the risks. To confirm these expectations, adverse events will be carefully monitored according to good clinical practice recommendations [26, 27]. If any, compensation to those who would suffer harm from trial participation would be covered by hospital insurance.

### Biases

Regarding selection biases, criteria of eligibility are clearly defined (Table 1) and were chosen to reflect the actual practice of surgical procedures for rib fixation. In addition, reasons for exclusion, as well as the number of subjects who decline participation, will be documented. A digital randomization procedure and allocation sequence concealment will be used.

Regarding other biases, validated questionnaires will be used to minimize interviewer bias. In addition, during follow-up, questionnaires will be collected at home via a web survey system. An intention-to-treat analysis allows to minimize bias introduced by exclusions or group changes.

For minimizing the introduction of biases due to missing data, a secure web application (REDCap) will be used for building and managing online surveys and study databases. Furthermore, data monitoring procedures (performed by external experienced monitors) and regular contact with the study’s participants (emails and phone calls) will be carried out.

### Study generalizability

This study is built as a pragmatic trial to perform it under normal conditions with the intention of providing results that are more applicable to clinical practice and decision-making. The use of three materials in the intervention group, the multiple possibilities to stabilize the chest wall and the use of a relevant primary outcome meaningful for participants are consistent to be generalizable.

### Trial status

The PAROS trial began recruiting in April 2021. The first patient was included in June 2021. The recruitment continues until April 2023.

## Conclusion

The PAROS trial will be the first clinical trial to compare pain in long term between surgical fixation and non-surgical treatment for uncomplicated rib fractures.

## Data Availability

Not applicable

## Abbreviations

PAROS: Pain after rib osteosynthesis
*HUG*: *Hôpitaux universitaires de Genève*
*CHUV*: *Centre hospitalier universitaire Vaudois*
INR: International normalized ratio
aPTT: Activated partial thromboplastin time
REDCap: Research Electronic Data Capture
NSAID: Non-steroidal anti-inflammatory drugs
BPI: Brief pain inventory
HADS: Hospital Anxiety and Depression Scale
*DN4*: *Douleur neuropathique en 4 questions*
SF-36: Short-form health survey 36
WPAI: Work productivity and activity impairment
PEF: Peak expiratory flow
ISO: International Organization for Standardization
ICH-GCP: International Council for Harmonization – good clinical practice
eCRF: Electronic case report form
SD: Standard deviation
IQR: Interquartile range
ANCOVA: Analysis of covariance
CI: Confidence interval
CRC: Clinical research center
SPIRIT: Standard Protocol Items: Recommendations for Interventional Trials
CONSORT: Consolidated Standards of Reporting Trials
